# Development and Initial Validation of the Disruptive Mood Dysregulation Disorder Questionnaire Among Adolescents From Clinic Settings

**DOI:** 10.3389/fpsyt.2022.617991

**Published:** 2022-02-17

**Authors:** Assia Boudjerida, Réal Labelle, Lise Bergeron, Claude Berthiaume, Jean-Marc Guilé, Jean-Jacques Breton

**Affiliations:** ^1^Department of Psychology, Université du Québec à Montréal, Montréal, QC, Canada; ^2^Centre for Research and Intervention on Suicide, Ethical Issues and End-of-Life Practices, Université du Québec à Montréal, Montréal, QC, Canada; ^3^Department of Psychiatry, Université de Montréal, Montréal, QC, Canada; ^4^Research Centre, Rivière-des-Prairies Mental Health Hospital, Centre Intégré Universitaire de Santé et de Services Sociaux du Nord-de-l'Île-de-Montréal, Université de Montréal, Montréal, QC, Canada; ^5^Department of Psychology, Université de Montréal, Montréal, QC, Canada; ^6^Department of Psychiatry, Université de Picardie Jules-Verne, Amiens, France

**Keywords:** adolescents, psychometric, disruptive mood dysregulation disorder, depression symptoms, borderline traits

## Abstract

**Objectives:**

Disruptive mood dysregulation disorder (DMDD) is a new DSM-5 diagnosis. It is observed in youths and is characterized by chronic irritability and temper outbursts. This study aimed (i) to develop a brief questionnaire administered during a semi-structured interview and (ii) to assess its psychometric properties with adolescents 12–15 years old by estimating its internal consistency and its concurrent association with measures of depressive symptoms and borderline personality traits.

**Methods:**

A 10-item questionnaire was developed based on the DSM-5 criteria and input from mental health professionals. The questionnaire was administered to 192 adolescents from youth centres, inpatient units and specialized outpatient clinics in Montreal, as were the Schedule for Affective Disorders and Schizophrenia for School-Aged Children (K-SADS-PL), the Abbreviated version of the Diagnostic Interview for Borderlines revised (Ab-DIB), and the Dominic Interactive for Adolescents-Revised (DIA-R).

**Results:**

A DMDD Questionnaire among adolescents from clinic settings is obtained. The content of the instrument's items was initially developed based on DSM-5 criteria and expert judgment to ensure that this new instrument covered the theoretical concepts of DMDD in English and French. Twelve participants (6.3%) met nine or more criteria and 11 youths (5.7%) met the three main criteria of DMDD (A, C, and D), which suggested the likely presence of DMDD. The total Cronbach's alpha was 0.90. In addition, the DMDD Questionnaire was significantly associated with depressive symptoms and borderline personality traits.

**Conclusion:**

The reliability and concurrent validity indices suggest that the questionnaire as a decision-support tool may be used with adolescents in clinical settings. It highlights that the DSM-5 DMDD criteria seem associated with depressive symptoms and borderline personality traits. Finally, future studies will be necessary to establish more robust calculations in relation to the validity and reliability of this questionnaire.

## Introduction

Disruptive mood dysregulation disorder (DMDD) is a condition characterized by chronic irritability observed in youths 6–18 years of age. Temper outbursts and emotional dysregulation are common reasons for seeking child and adolescent psychiatric and psychological consultations. However, what sets DMDD apart is the frequency and severity of the outbursts (at least three times a week) and the persistence of negative affect practically all day and every day (1). The American Psychiatric Association (2) classified DMDD as a depressive disorder and has indicated that it affects 2–5% of children and adolescents in the general population. For a diagnosis to be made, all of the DSM-5 criteria listed in Table 1 must be present.

**Table 1:** DSM-5 diagnostic criteria for DMDD and DMDD Questionnaire items.

**DSM-5 criteria**	**Item number**	**Description of diagnostic criterion and item**
A_1_	1	Severe recurrent temper outbursts manifested verbally and/or behaviourally.
A_2_	2	These outbursts are grossly out of proportion in intensity or duration to the situation or provocation.
B	Not assessed	The temper outbursts are inconsistent with developmental level.
C	3	The temper outbursts occur, on average, three or more times per week.
D_1_	4	The mood between temper outbursts is persistently irritable or angry most of the day, nearly every day.
D_2_	5	This mood is observable by others.
E_1_	6	Criteria A–D have been present for 12 or more months.
E_2_	7	There has not been a period lasting three or more consecutive months without all of the symptoms in Criteria A–D.
F_1_	8	Criteria A and D are present in at least two of three settings (at home, at school, with peers).
F_2_	10	These criteria are severe in at least one of these settings.
G	Assessed pre-administration	The diagnosis should not be made for the first time before age 6 years or after age 18 years (condition met by virtue of age of target client group)
H	9	The age of onset of Criteria A–E is before 10 years.
I	Not assessed	Exclusion criterion: presence of all the symptoms of a manic or hypomanic episode for more than 1 day.
J	Not assessed	Symptoms not better explained otherwise.

*This diagnosis cannot co-exist with oppositional defiant disorder, intermittent explosive disorder, or bipolar disorder*.

A link has been observed between DMDD and unipolar depression. Young people with DMDD generally develop unipolar depressive disorders or anxiety disorders as they move through adolescence into adulthood (3–5). In this regard, Copeland et al. (6) noted a co-occurrence between DMDD and depression among young people 2–17 years old (odds ratios between 9.9 and 23.5). Besides, the relationship between DMDD and borderline personality traits as defined under the DSM-5 has yet to be investigated in adolescents. This link makes sense considering the central role of emotional dysregulation suggested by the biosocial model of the development of borderline personality (7). Although this model does not refer directly to the concept of DMDD, hypersensitivity and intense reactions to emotional stimuli are key components of this personality disorder. In this regard, Glenn and Klonsky (8) observed a significant association (*r* = 0.54) between a measure of emotional dysregulation and borderline personality traits among young adults. In short, it would be interesting to explore the relationship between DMDD and, respectively, depressive symptoms and borderline personality traits to reflect on the matter further.

Furthermore, Mürner-Lavanchy, Kaess (9) recently published a systematic review of existing measures of DMDD. They noted that there was no gold standard for assessing the disorder. However, the authors indicated that the DMDD module created in 2016 by Kaufman, Birmaher (10) included in the Kiddie Schedule for Affective Disorders and Schizophrenia for School-Aged Children (K-SADS-PL) had been used in 25% of the studies of DMDD, making it the most popular measure to date. To our knowledge, however, only one study measured the module's validity. In fact, Unal, Oktem (11) examined the concurrent validity between a clinical psychiatric interview based on DSM-5 diagnostic criteria (κ = 0.70) and the Turkish version of the K-SADS-PL (κ = 0.63). Consequently, we cannot consider the DMDD module of the K-SADS-PL a validated measure solely on this basis. Moreover, the K-SADS-PL remains a time-consuming instrument used mainly for research purposes. Consequently, it would be useful to have a short clinical decision-support instrument, based on the DSM-5 criteria for DMDD, for use before or during a classic psychiatric evaluation.

It need be underscored, also, that DMDD studies to date have focused on psychometric instruments completed by parents (12–15). According to Achenbach, McConaughy (16), however, there exists a reporting bias associated with children's informants (parents, peers, teachers). Examining the answers given by mothers and their children 6–23 years old in the context of the latter's psychiatric evaluation, Weissman (17) found that the former tended to underestimate symptoms, compared with the latter. Other researchers have specified that such underestimation occurred primarily when children presented symptoms of internalizing disorders (18). This is why some authors have suggested that, with children 10 years and over, instruments based on child and adolescent report should be included as part of their psychological evaluation (16, 19, 20). Consequently, it would be useful to develop a questionnaire for assessing DMDD symptoms reported by adolescents themselves in addition to one completed by their legal guardians.

Against this background, we undertook a study aimed at further developing the DMDD Questionnaire and assessing its psychometric properties among adolescents 12–15 years old from clinical settings. From a psychometric point of view, this is the first step in the validation of a decision-support tool for screening adolescents for DMDD (21, 22). Two objectives were formulated: (1) to develop a brief questionnaire administered during a semi-structured interview and (2) to assess its initial psychometric properties with adolescents 12–15 years old by estimating its internal consistency and its concurrent association with measures of depressive symptoms and borderline personality traits.

## Materials and Methods

### Participants

The DMDD Questionnaire was administered to adolescents in Montreal from 2011 to 2014 as part of a cross-sectional study of the psychometric properties of the French and English versions of the Dominic Interactive for Adolescents–Revised (DIA-R). The initial sampling plan of this study aimed at recruiting a sufficiently large convenience sample, which included a school subsample and a clinic subsample, to obtain accurate estimates to determine the instrument's reliability by age, sex and language subgroups and its criterion-related validity (20). Adolescents had to meet two inclusion criteria to participate: be 12–15 years old and speak French or English. The respondent parent, too, had to understand and speak French or English to complete the ethical consent form. Sight- and hearing-impaired adolescents were excluded, as were those with severe intellectual or learning disabilities (20). The sample comprised 447 adolescents living in the Greater Montreal Area: 243 adolescents (130 French speaking, 113 English speaking) selected in regular classrooms at four high schools reflecting a wide array of socioeconomic levels and 204 adolescents (171 French speaking, 33 English speaking) from two youth centres and specialized psychiatric clinics, inpatient units, and day treatment centres at three hospitals. These clinical settings provided services for adolescents from families with different cultural and socioeconomic backgrounds. Because the DMDD Questionnaire was designed for clinical purposes, we ran statistical analyses only on the subsample of adolescents recruited in clinical settings. Participants with missing data (*n* = 12) were excluded. As a result, the convenience sample considered in the analyses consisted of 192 adolescents. The percentages of adolescents by age, sex and language subgroups remained quite similar after these 12 were excluded (see descriptive statistics).

### Primary Measure: DMDD Questionnaire

**Figure 1:**
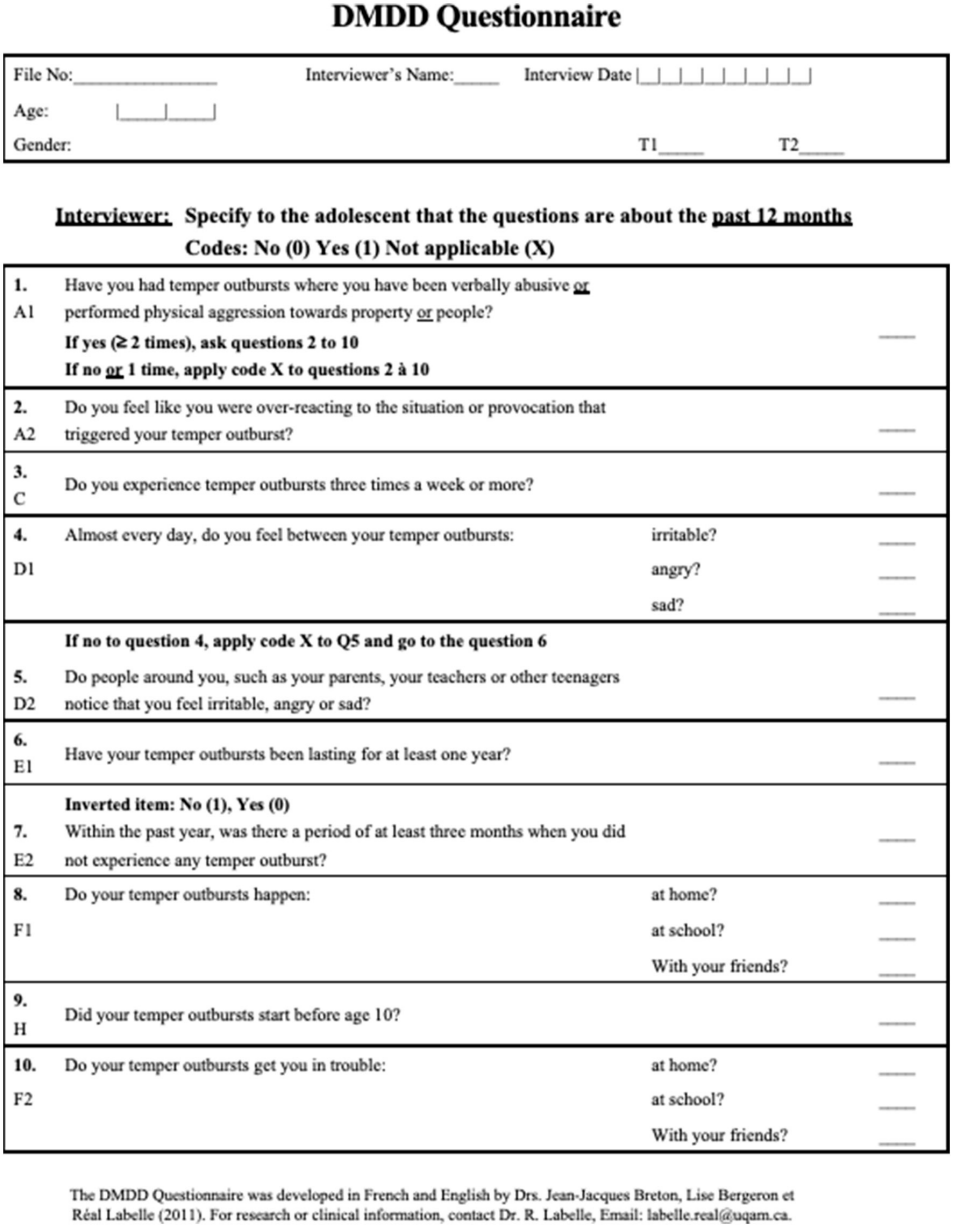
DMDD Questionnaire.

The *DMDD Questionnaire* (Figure 1) was developed at the Research Centre of the Rivière-des-Prairies Mental Health Hospital. As shown in Table 1, work regarding the questionnaire's content validity led to the creation of an algorithm to establish correspondence between the questionnaire's items and some of the DSM-5 criteria (A, C, D, E, F, G, and H). It should be noted that some criteria were split in two so that questions could be as simple as possible. It should be noted, also, that exclusion criteria were omitted, namely, criteria B, I, and J, for the sake of brevity. The DMDD Questionnaire is composed of 10 yes/no questions. If the answer to the first question is “yes,” then the nine other questions are asked. However, if the answer to the first question is “no,” the subsequent questions are not completed and “no” is indicated throughout (except at question 7, which is an inverted item). Thus, the DMDD Questionnaire, which covers seven DSM-5 criteria, yields a continuous score ranging from 0 to 10. Each yes (except for the inverted item 7) adds a point to the total. The higher the score, the higher the likelihood of DMDD.

### Reference Measures

The Schedule for Affective Disorders and Schizophrenia for School-Aged Children (K-SADS-PL) is a semi-structured interview widely used in psychiatry to assess the mental disorders most common among 7- to 17-year-olds. Given that the DSM-5 version of the instrument with the DMDD module was not yet available at the time of our study (10), the *Present and Lifetime* version (1997) of the child-report instrument was used (23). However, changes proposed by the DSM-5 were taken into account during interviews (20). The instrument possesses moderate-to-high test-retest reliability (K = 0.63–0.90) and almost perfect inter-rater reliability (93–100%) (23). In the psychometric study of the DIA-R, the English and French versions of the K-SADS-PL were used to assess the adolescents' perception of their symptoms for nine disorders in the past 6 months. In our study, we examined the relationship between the DMDD Questionnaire and depression based on the criteria score.

The Abbreviated version of the Diagnostic Interview for Borderlines revised (Ab-DIB) is a 26-item self-report questionnaire for examining borderline personality traits in the past year. Scores range from 0 to 52. Its reliability and criterion validity have been investigated among suicidal adolescents 14–17 years old (24). Reliability coefficients were ≥0.80. Compared with the Diagnostic Interview for Borderlines–Revised, the Ab-DIB demonstrated an AUC of 0.87 (24). Although the Ab-DIB was previously used with older adolescents, preliminary analyses in the main study of the DIA-R yielded alpha coefficients ≥0.80 for all age (12–13 years, 14–15 years), sex, and language subgroups (20).

The Dominic Interactive for Adolescents–Revised (DIA-R) is a 121-item pictorial computerized self-report questionnaire for adolescents based on DSM-5 diagnostic criteria (20). It allows clinicians to screen for major mental health problems, including depressive symptoms and borderline personality traits, based on symptom and criteria scores. The color pictures present Dominic as a function of the respondent's sex and ethnicity (Caucasian, Hispanic, African American, or Asian). Adolescents respond by clicking “Yes” or “No” on the screen. For the total sample, Cronbach alpha coefficients were >0.80 for the major depressive disorder scale and ≥0.75 for the borderline personality traits scale (20). Moreover, for the total sample, the test-retest estimates of reliability (ICCs) ranged from 0.75 to 0.94 for specific scales. Regarding the criterion-related validity, ROC analyses were completed in the course of the main study. The AUCs for the major depressive disorder scale and for the borderline personality traits scale were both 0.91 (20).

### Ethical Considerations

The Institutional Review Board of the Rivière-des-Prairies Mental Health Hospital (CIUSSS du Nord-de-l'Île-de-Montréal) approved the research protocol of this study. All participants and their parents gave their written informed consent.

### Statistical Analyses

All analyses were run on the IBM SPSS Statistics 23 application. First, descriptive analyses were carried out to see how the sample was distributed over the questionnaire scores and criteria. Then, Cronbach's alpha coefficient (25) and its confidence intervals (26) were calculated to verify the internal consistency of the DMDD Questionnaire (22, 27). A coefficient ≥0.70 suggests acceptable internal consistency (28, 29). Finally, Pearson's correlation coefficients were calculated between the DMDD score and the scores obtained for depressive symptoms and borderline personality traits. The statistical power test showed that these analyses reached the recommended power (80%) to detect medium or large effects as defined by Cohen (30).

## Results

### First Objective

The DMDD Questionnaire was developed in 2011 by Breton, Bergeron and Labelle (31). Their objective was to construct a decision-support tool for the psychiatric evaluation of children with both behavioral and emotional symptoms. Moreover, they wanted to collect data on this new and controversial diagnosis. They took the criteria proposed by the DSM-5 Task Force and formulated them in the form of simple questions to be put directly to adolescents during an interview. The questionnaire comprises ten “yes/no” questions. It may be administered by a nurse, a psychologist or a trained research assistant and allows the interviewer to clarify time frames and provide synonyms and examples, if needed. The questions were initially drafted in English by Breton using the same wording as that used by the Task Force in their criteria. Then, Breton, Bergeron and Labelle (31) each drafted and revised a French version and a consensus was reached on the final formulation of each question. Finally, a professional translator was tasked with verifying the equivalence between the French and the English versions. This questionnaire has been presented in Figure 1.

### Second Objective

#### Descriptive Statistics

The clinic sample was composed of 56 youths (29.2%) 12 or 13 years old and 136 youths (70.8%) 14 or 15 years old. There were more girls (55.2%) than boys (44.8%) and more French speakers (85.4%) than English speakers (14.6%). In this sample, 41.7% of the adolescents answered “no” to the first question on the questionnaire and therefore did not complete the remaining questions. Conversely, two adolescents (1%) met all the criteria measured by the DMDD Questionnaire and obtained a score of 10/10. Twelve participants (6.3%) met nine or more criteria and 11 (5.7%) met the three main criteria of DMDD (A, C and D), which suggested the likely presence of DMDD.

Frequencies for the DMDD Questionnaire items are given for presence (criteria A, C, and D) and severity (criteria E and H) of symptoms and for adaptation problems in different settings (criterion F). The results presented in Table 2 show that few youths (5.7%) presented the key DMDD symptoms (criteria A, C, and D). As expected, when the number of criteria to be met increased, the percentage of youths that met all of them fell. In this regard, the addition of the timing criterion (criteria E and H) resulted in a considerable drop in the number of youths that did so.

**Table 2:** Frequency of DMDD questionnaire items based on DSM-5 criteria.

**DSM-5 criteria**	**Convenience sample**	
	**(*****n*** **=** **192)**	
	***n***	**%**
A_1, 2_, C, D_1, 2_ intense temper outbursts three times a week and irritable mood in between	11	5.7
A_1, 2_, C, D_1, 2_ and E_1, 2_ symptoms present for a year with no asymptomatic period	4	2.1
A_1, 2_, C, D_1, 2_, E_1, 2_ and H onset of symptoms before age 10	2	1
A_1, 2_, C, D_1, 2_, E_1, 2_, H et F_1, 2_ symptoms present and causing problems in different settings	2	1

*Frequency for each criterion. A1, 58.3%; A2, 37.0%; C, 13.0 %; D1irr, 24.5%; D1ang, 21.4%; D1sad, 20.3%; D2, 42.7%; E1, 45.3%; E2, 26.6%; F1hom, 54.2%; F1sch, 24.0%; F1fri =15.6%; H, 25.0%; F2hom, 44.3%; F2sch, 17.2%; F2fri, 8.9%*.

#### Internal Consistency

The internal consistency of the construct was the only index of reliability of the DMDD Questionnaire measured. The alpha was 0.90 for this sample and the 95% confidence intervals are 0.88–0.92.

#### Concurrent Validity

The association between the continuous measure of DMDD and the other continuous measures of mental health problem was assessed for the sample. First, the correlation coefficients revealed a significant link between DMDD and, respectively, depressive symptoms (*r* = 0.310, *p* = 0.001 for the DIA-R and *r* = 0.144, *p* = 0.049 for the K-SADS-PL) and borderline personality traits (*r* = 0.427, *p* = 0.001 for the DIA-R and *r* = 0.261, *p* = 0.001 for the Ab-DIB).

## Discussion

This is the first study of a brief questionnaire that allows obtaining information from adolescents regarding the principal DMDD criteria. The content of the instrument's items was initially developed based on DSM-5 criteria and expert judgment to ensure that this new instrument covered the theoretical concepts of DMDD in English and French. The reliability and concurrent validity indices suggest that the questionnaire may be used in a clinical context.

In addition, our results show that DMDD is relatively rare. Overall, 12 participants or 6.3% of the sample scored at least nine out 10 on the questionnaire, and almost as many met criteria A, C and D (5.7%). These figures fall within the prevalence estimated in the general population according to the DSM-5 (2–5%) (2) but are lower than those usually observed in clinical settings (13, 32, 33) probably due to differences in DMDD measures (9, 34). When time criteria were added (symptoms present for a year with no asymptomatic period of more than 3 months), the percentage dropped to 2.1%. We therefore suggest to clinicians who might use this questionnaire to suspect the presence of DMDD if the respondent scores nine or more or answers “yes” on the items regarding the symptoms of DMDD (criteria A, C, D, and questions 1–5).

In general, this first step in the validation of the DMDD Questionnaire shows that the instrument possesses satisfactory initials psychometric properties. The construct's internal consistency is the questionnaire's only reliability index. From an interpretative perspective, the questionnaire's structure, which includes a main question (Q1) and nine contingency items, is largely conducive to the high degree of consistency observed relative to dimensional scales without this contingency. However, it is important to underscore that the alpha coefficient obtained (0.90) suggests an acceptable internal consistency (>0.70) (27). It should also be noted that some authors raise few limitations associated with the use of sum scores as in the calculation of Cronbach's alpha (35, 36). It is however possible to use sum scores insofar as a factorial analysis carried out in the preliminary analyzes of this study showed that a single factor is present in the DMDD Questionnaire. Also, since it is a screening tool and not an accurate diagnostic tool, it seems acceptable to use the sum score in this initial validation study (35). Being aware of all these limitations, we cannot reach a firm conclusion regarding internal consistency. Still, these initial results push us toward a future study that will allow us to do a more solid validation on another sample.

The results regarding concurrent validity to examine the relationship between the DSM-5 DMDD criteria and two related constructs suggests that the DMDD criteria in adolescence are significantly associated with depressive symptoms and borderline personality traits. Classifying DMDD in the DSM-5 category of depressive disorders reflects the fact that youths that present these symptoms generally develop depressive or anxiety disorders as they approach adulthood (3–5). Additionally, part of the convergence between DMDD and depression can also be explained by the fact that irritability and negative mood are symptoms of depression that are also found in youth (2). The significant correlation between the DSM-5 DMDD criteria and borderline personality traits is interesting as well. Perepletchikova, Nathanson (37) hypothesized a link between these two constructs, noting that the two disorders shared the core element of emotional dysregulation. This hypothesis enabled these authors to develop a treatment based on dialectical behavior therapy for DMDD. Moreover, Guilé, Boissel (38) reported that the presence of externalizing disorders in childhood predicted borderline personality traits in early adolescence, whereas depression in adolescence predicted borderline personality traits in adulthood. This concurs with the portrait of DMDD, namely, childhood marked by excessive temper outbursts and adolescence marked by depressive symptoms. Hence, it is reasonable to think that the trajectory proposed by these authors applies here. Especially since our research on the DMDD Questionnaire indicates a possible association between DMDD, depressive symptoms, and borderline personality traits in adolescence. Future research should validate this hypothesis. Studying the relationship between these concepts could help steer the treatment options for these youths.

These findings also have practical implications. From a clinical viewpoint, the questionnaire is useful in that it takes little time to administer. It is all the more useful since the rates of comorbidity in DMDD are high (2). A decision support tool is therefore relevant. Furthermore, the items are put directly to the adolescent in English or French. In addition, compared with the DMDD module of the K-SADS-PL (10), the DMDD Questionnaire proposes a small number of clear, easy-to-understand items regarding DMDD alone. What's more, the score yielded by the questionnaire gives an idea of the number of symptoms and timing elements that correspond to the diagnostic criteria. The instrument thus makes a contribution above and beyond the K-SADS's utility.

Our study has limitations. First, the DMDD Questionnaire is a decision-support instrument and, by definition, cannot serve as the basis for rendering a psychiatric diagnosis. It could serve as a brief questionnaire administered prior to a complete psychiatric evaluation. Second, this exploratory study represents a first step in the psychometric validation of the instrument. While the criteria selected remain pertinent, the absence of another measure to evaluate DMDD based on DSM-5 criteria restricts the possibility of comparing the questionnaire against an external validation criterion that refers to this construct. Once again, we have to keep in mind that the K-SADS-PL with the DMDD module was published after our study was carried out (10). Third, the initial study design did not allow evaluating the questionnaire's test-retest reliability. Fourth, we did not use or develop a version of the DMDD Questionnaire for parents. Fifth, the instrument's comprehensibility of some questions was not examined. Sixth, the convenience sample was not representative of all adolescents with DMDD symptoms from the clinical population. The absence of representativeness limits the extent to which we can generalize the results to this population. Finally, although the results suggest a possible relationship between DMDD criteria and depressive symptoms and borderline personality traits, the correlation coefficients remain modest, the level varying from low to moderate (see Table 3). Note that although the correlations are present, some might say that there is a reasonable doubt as to whether they are real (39). Future studies will be necessary to establish more robust calculations in relation to the validity and reliability of this questionnaire.

**Table 3:** Pearson's correlations for concurrent validity.

**Criterion score**	**Symbols**	**DIA-R depression symptoms**	**K-SADS depression symptoms**	**DIA-R borderline traits**	**Ab-DIB borderline traits**
	*r* coefficient	0.310^**^	0.144^*^	0.427^**^	0.261^**^
	Sig. (two-tailed)	0.001	0.049	0.001	0.001
	*N*	192	187	192	191
Confidence interval	Lower	0.176	−0.004	0.298	0.123
	Upper	0.440	0.281	0.525	0.397

*r = 0.10 is a weak correlation; r = 0.30 is a moderate correlation; r = 0.50 is a strong correlation. 95% bootstrap confidence intervals*.

** *Correlation significant at 0.01 (two-tailed);*

* *Correlation significant at 0.05 (two-tailed)*.

In summary, the results suggest that the DMDD Questionnaire presents adequate initial psychometric properties when used with adolescents from clinical settings. The results allow clinicians and researchers to use a practical, brief questionnaire based on DSM-5 criteria as a decision-support tool. Finally, this study supports the presence of association between the DSM-5 DMDD criteria and depressive symptoms and, to our knowledge, this is the first study to show that DMDD could be associated with borderline personality traits.

## Data Availability Statement

The raw data supporting the conclusions of this article will be made available by the authors, without undue reservation.

## Ethics Statement

The studies involving human participants were reviewed and approved by Institutional Review Board of the Rivière-des-Prairies Mental Health Hospital (CIUSSS du Nord-de-l'Île-de-Montréal). Written informed consent to participate in this study was provided by the participants' legal guardian/next of kin.

## Author Contributions

RL, LB, and J-JB contributed to the conception and design of the DMDD Questionnaire and the study. CB organized the database and performed the statistical analysis. J-MG helped interpret the data. AB wrote the first draft of the manuscript as part of her thesis. RL and LB drafted sections of the manuscript. All authors contributed to the manuscript's revision and read and approved the submitted version. The Canadian French and English versions of the DMDD Questionnaire are available for use from RL.

## Funding

The authors would like to thank the Research Centre of the Rivière-des-Prairies Mental Health Hospital (CIUSSS du Nord-de-l'Île-de-Montréal) and the Centre for Research and Intervention on Suicide, Ethical Issues and End-of-Life Practices (Université du Québec à Montréal) for their financial support.

## Conflict of Interest

The authors declare that the research was conducted in the absence of any commercial or financial relationships that could be construed as a potential conflict of interest.

## Publisher's Note

All claims expressed in this article are solely those of the authors and do not necessarily represent those of their affiliated organizations, or those of the publisher, the editors and the reviewers. Any product that may be evaluated in this article, or claim that may be made by its manufacturer, is not guaranteed or endorsed by the publisher.

## References

[1] Fristad MA Wolfson H Algorta GP Youngstrom EA Arnold LE Birmaher B. Disruptive mood dysregulation disorder and bipolar disorder not otherwise specified: fraternal or identical twins? J Child Adol Psychopharmacol. (2016) 26:138–46. 10.1089/cap.2015.0062 26859630 PMC4800383

[2] American Psychiatric Association. Diagnostic and Statistical Manual of Mental Disorders DSM-5. 5th ed. Arlington, VA: American Psychiatric Publishing (2013).

[3] Brotman MA Schmajuk M Rich BA Dickstein DP Guyer AE Costello EJ. Prevalence, clinical correlates, and longitudinal course of severe mood dysregulation in children. Biol Psychiatry. (2006) 60:991–7. 10.1016/j.biopsych.2006.08.042 17056393

[4] Copeland WE Shanahan L Egger H Angold A Costello EJ. Adult diagnostic and functional outcomes of DSM-5 disruptive mood dysregulation disorder. Am J Psychiatry. (2014) 171:668–74. 10.1176/appi.ajp.2014.13091213 24781389 PMC4106474

[5] Stringaris A Cohen P Pine DS Leibenluft E. Adult outcomes of youth irritability: a 20-year prospective community-based study. Am J Psychiatry. (2009) 166:1048–54. 10.1176/appi.ajp.2009.08121849 19570932 PMC2791884

[6] Copeland WE Angold A Costello EJ Egger H. Prevalence, comorbidity, and correlates of DSM-5 proposed disruptive mood dysregulation disorder. Am J Psychiatry. (2013) 170:173–9. 10.1176/appi.ajp.2012.12010132 23377638 PMC3573525

[7] Crowell SE Beauchaine TP Linehan MM. A biosocial developmental model of borderline personality: elaborating and extending Linehan's theory. Psychol Bull. (2009) 135:495–510. 10.1037/a0015616 19379027 PMC2696274

[8] Glenn CR Klonsky ED. Emotion dysregulation as a core feature of borderline personality disorder. J Person Dis. (2009) 23:20–8. 10.1521/pedi.2009.23.1.20 19267659

[9] Mürner-Lavanchy I Kaess M Koenig J. Diagnostic instruments for the assessment of disruptive mood dysregulation disorder: a systematic review of the literature. Eur Child Adol Psychiatry. (2021) 2021:1–23. 10.1007/s00787-021-01840-4 34232390 PMC9908712

[10] Kaufman J Birmaher B Axelson D Perepletchikova F Brent D Ryan N. Schedule for Affective Disorders and Schizophrenia for School-Aged Children: Present and Lifetime Version (K-SADS-PL) DSM-5. New Haven: Yale University. Child and Adolescent Research and Education (2016).

[11] Unal F Oktem F Cetin Cuhadaroclu F Cengel Kultur SE Akdemir D Foto Ozdemir D. Reliability and validity of the schedule for affective disorders and schizophrenia for school-age children-present and lifetime version, DSM-5 November 2016-Turkish adaptation (K-SADS-PL-DSM-5-T). Turk Psikiyatri Derg. (2019) 30:42–50. 10.5080/u23408 31170306

[12] Dougherty LR Smith VC Bufferd SJ Carlson GA Stringaris A Leibenluft E. DSM-5 disruptive mood dysregulation disorder: correlates and predictors in young children. Psychol Med. (2014) 44:2339–50. 10.1017/S0033291713003115 24443797 PMC4480202

[13] Margulies DM Weintraub S Basile J Grover PJ Carlson GA. Will disruptive mood dysregulation disorder reduce false diagnosis of bipolar disorder in children? Bipolar Dis. (2012) 14:488–96. 10.1111/j.1399-5618.2012.01029.x 22713098

[14] Mayes SD Waxmonsky JD Calhoun SL Bixler EO. Disruptive mood dysregulation disorder symptoms and association with oppositional defiant and other disorders in a general population child sample. J Child Adol Psychopharmacol. (2016) 26:101–6. 10.1089/cap.2015.0074 26745442 PMC4800381

[15] Laporte PP Matijasevich A Munhoz TN Santos IS Barros AJ Pine DS. Disruptive mood dysregulation disorder: symptomatic and syndromic thresholds and diagnostic operationalization. J Am Acad Child Adol Psychiatry. (2021) 60:286–95. 10.1016/j.jaac.2019.12.008 32004697 PMC9073144

[16] Achenbach TM McConaughy SH Howell CT. Child/adolescent behavioral and emotional problems: implications of cross-informant correlations for situational specificity. Psychol Bull. (1987) 101:213. 10.1037/0033-2909.101.2.213 3562706

[17] Weissman MM Wickramaratne P Warner V John K Prusoff BA Merikangas KR. Assessing psychiatric disorders in children: discrepancies between mothers' and children's reports. Arch Gen Psychiatry. (1987) 44:747–53. 10.1001/archpsyc.1987.01800200075011 3632247

[18] Rothen S Vandeleur CL Lustenberger Y Jeanprêtre N Ayer E Gamma F. Parent–child agreement and prevalence estimates of diagnoses in childhood: direct interview versus family history method. Int J Meth Psychiatric Res. (2009) 18:96–109. 10.1002/mpr.281 19507167 PMC6878311

[19] Bergeron L Smolla N Valla J-P St-Georges M Berthiaume C Piché G. Psychometric properties of a pictorial instrument for assessing psychopathology in youth aged 12 to 15 years: the dominic interactive for adolescents. Can J Psychiatry. (2010) 55:211–21. 10.1177/070674371005500404 20416144

[20] Bergeron L Smolla N Berthiaume C Renaud J Breton J-J St.-Georges M. Reliability, validity, and clinical utility of the dominic interactive for adolescents–revised: a DSM-5–based self-report screen for mental disorders, borderline personality traits, and suicidality. Can J Psychiatry. (2017) 62:211–22. 10.1177/0706743716670129 27638424 PMC5317018

[21] Anastasi A. Psychological Testing. 6e ed. New York, NY: Macmillan Publishing Co, Inc (1988).

[22] Urbina S. Essentials of Psychological Testing. Hoboken, NJ: John C Wiley & Sons. Inc (2004).

[23] Kaufman J Birmaher B Brent D Rao U Flynn C Moreci P. Schedule for affective disorders and schizophrenia for school-aged children: present and lifetime version (K-SADS-PL): initial reliability and validity data. J Am Acad Child Adol Psychiatry. (1997) 36:980–8. 10.1097/00004583-199707000-00021 9204677

[24] Guilé J-M Greenfield B Berthiaume C Chapdelaine C Bergeron L. Reliability and diagnostic efficiency of the abbreviated-diagnostic interview for borderlines in an adolescent clinical population. Eur Child Adol Psychiatry. (2009) 18:575–81. 10.1007/s00787-009-0015-x 19390770

[25] Cronbach LJ. Coefficient alpha and the internal structure of tests. Psychometrika. (1951) 16:297–334. 10.1007/BF02310555

[26] Duhachek A Iacobucci D. Alpha's standard error (ASE): an accurate and precise confidence interval estimate. J Appl Psychol. (2004) 89:792. 10.1037/0021-9010.89.5.792 15506861

[27] Streiner DL Norman GR Cairney J. Health Measurement Scales: A Practical Guide to Their Development and Use. New York, NY: Oxford University Press (2015).

[28] Cicchetti DV. Guidelines, criteria, and rules of thumb for evaluating normed and standardized assessment instruments in psychology. Psychol Assess. (1994) 6:284–90. 10.1037/1040-3590.6.4.284

[29] Ponterotto JG Ruckdeschel DE. An overview of coefficient alpha and a reliability matrix for estimating adequacy of internal consistency coefficients with psychological research measures. Percept Motor Skills. (2007) 105:997–1014. 10.2466/pms.105.3.997-1014 18229554

[30] Cohen J. Statistical Power Analysis for the Behavioral Sciences. New York, NY: Academic Press, Inc (2013).

[31] Breton J-J Bergeron L Labelle R. DMDD Questionnaire (Questionnaire du TDDE). Montréal, QC: Laboratory of Mood Disorders (2011).

[32] Axelson D Findling RL Fristad MA Kowatch RA Youngstrom EA Horwitz SM. Examining the proposed disruptive mood dysregulation disorder diagnosis in children in the longitudinal assessment of manic symptoms study. J Clin Psychiatry. (2012) 73:1342–50. 10.4088/JCP.12m07674 23140653 PMC3581334

[33] Freeman AJ Youngstrom EA Youngstrom JK Findling RL. Disruptive mood dysregulation disorder in a community mental health clinic: prevalence, comorbidity and correlates. J Child Adol Psychopharmacol. (2016) 26:123–30. 10.1089/cap.2015.0061 26745325 PMC4800380

[34] Carlson GA. Disruptive Mood Dysregulation Disorder. In: Long-term Outcomes in Psychopathology Research: Rethinking the Scientific Agenda. New York, NY: Oxford University Press (2015). p. 103–22.

[35] McNeish D Wolf MG. Thinking twice about sum scores. Behav Res Meth. (2020) 52:2287–305. 10.3758/s13428-020-01398-0 32323277

[36] McNeish D. Thanks coefficient alpha, we'll take it from here. Psychol Meth. (2018) 23:412. 10.1037/met0000144 28557467

[37] Perepletchikova F Nathanson D Axelrod SR Merrill C Walker A Grossman M. Randomized clinical trial of dialectical behavior therapy for preadolescent children with disruptive mood dysregulation disorder: feasibility and outcomes. J Am Acad Child Adol Psychiatry. (2017) 56:832–40. 10.1016/j.jaac.2017.07.789 28942805

[38] Guilé J-M Boissel L Alaux-Cantin S de La Rivière SG. Borderline personality disorder in adolescents: prevalence, diagnosis, and treatment strategies. Adol Health Med Therap. (2018) 9:199. 10.2147/AHMT.S156565 30538595 PMC6257363

[39] Meehl PE. Why summaries of research on psychological theories are often uninterpretable. Psychol Rep. (1990) 66:195–244. 10.2466/pr0.1990.66.1.195

